# A review of the effects of unilateral hearing loss on spatial hearing

**DOI:** 10.1016/j.heares.2018.08.003

**Published:** 2019-02

**Authors:** Daniel P. Kumpik, Andrew J. King

**Affiliations:** Department of Physiology, Anatomy and Genetics, Parks Road, Oxford, OX1 3PT, UK

**Keywords:** Sound localization, Spatial release from masking, Plasticity, Binaural, Monaural spectral cue, Auditory cortex

## Abstract

The capacity of the auditory system to extract spatial information relies principally on the detection and interpretation of binaural cues, i.e., differences in the time of arrival or level of the sound between the two ears. In this review, we consider the effects of unilateral or asymmetric hearing loss on spatial hearing, with a focus on the adaptive changes in the brain that may help to compensate for an imbalance in input between the ears. Unilateral hearing loss during development weakens the brain's representation of the deprived ear, and this may outlast the restoration of function in that ear and therefore impair performance on tasks such as sound localization and spatial release from masking that rely on binaural processing. However, loss of hearing in one ear also triggers a reweighting of the cues used for sound localization, resulting in increased dependence on the spectral cues provided by the other ear for localization in azimuth, as well as adjustments in binaural sensitivity that help to offset the imbalance in inputs between the two ears. These adaptive strategies enable the developing auditory system to compensate to a large degree for asymmetric hearing loss, thereby maintaining accurate sound localization. They can also be leveraged by training following hearing loss in adulthood. Although further research is needed to determine whether this plasticity can generalize to more realistic listening conditions and to other tasks, such as spatial unmasking, the capacity of the auditory system to undergo these adaptive changes has important implications for rehabilitation strategies in the hearing impaired.

## Highlights

• Unilateral hearing loss in infancy can disrupt spatial hearing, even after binaural inputs are restored.
• Plasticity in the developing brain enables substantial recovery in sound localization accuracy.
• Adaptation to unilateral hearing loss is based on reweighting of monaural spectral cues and binaural plasticity.
• Training on auditory tasks can partially compensate for unilateral hearing loss, highlighting potential therapies.

## 1 Introduction

An ability to localize and segregate different sound sources is extremely important for most species that can hear, often playing a crucial role in guiding behavioral responses, such as seeking out potential mates or prey or avoiding and escaping from approaching predators. This is particularly the case if the source lies beyond the detection range of the other senses, either because it is located outside the visual field or is too far away to be registered by other sensory receptors. The basis for directional hearing relies principally on the fact that animals have two ears that are physically separated on either side of the head, or in the case of some insects, on other parts of the body. This means that, depending on the location of the sound source, the signals reaching each ear may differ in their time of arrival or intensity, giving rise to binaural spatial cues (reviewed by Blauert, 1997, Schnupp et al., 2011).

A large number of studies have demonstrated the importance of binaural cues for sound-source localization, as well as for improving the perception of target sounds in the presence of other, interfering sounds (Bronkhorst, 2000), and a great deal is known about how these cues are processed in the brain (reviewed by Grothe et al., 2010). By eliminating binaural cues, or at least altering the relationship between the interaural acoustic differences and directions in space, unilateral or asymmetric hearing loss can have very disruptive effects on spatial hearing. Furthermore, monaural deprivation in infancy can induce maladaptive changes in the brain that may persist even if hearing in the affected ear is restored, resulting in longer-term deficits in spatial hearing (Kaplan et al., 2016). However, as described in the following sections, there is growing evidence that the plasticity of central auditory processing can help to partially compensate for loss of hearing in one ear, leading to some recovery in the ability to localize sound (e.g. Knudsen et al., 1984, Keating et al., 2013, Keating et al., 2015, Keating et al., 2016). In this article, we review the effects of asymmetric hearing loss on spatial processing, both during development and in later life, and consider the factors that may promote adaptive changes in the brain and their potential clinical relevance.

## 2 The importance and limitations of binaural processing

**Fig. 1:**
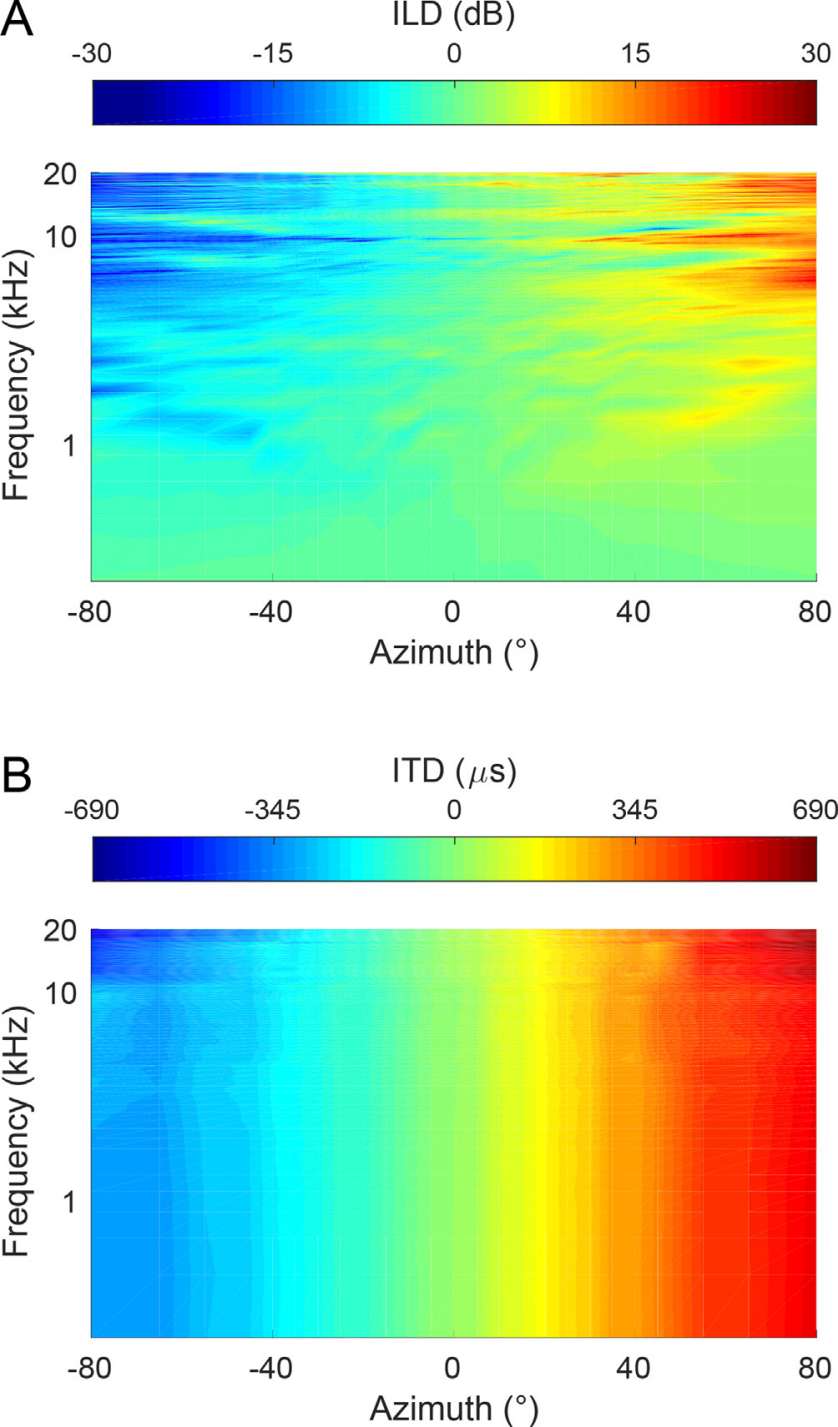
Binaural cues to sound source location. (A) Interaural level differences as a function of sound azimuth and frequency. (B) Interaural time differences as a function of sound azimuth and frequency. Negative values indicate azimuths and corresponding binaural cue values on the left of the midline. Data for both cues are derived from head-related transfer function measurements (0° elevation) published in the CIPIC database by Algazi et al. (2001). (Copyright (c) 2001 The Regents of the University of California. All Rights Reserved).

Because cochlear hair cells are tuned to different sound frequencies, with their topographically organized outputs producing tonotopic maps throughout the core or lemniscal regions of the central auditory pathway, sound-source location has to be computed through the sensitivity of neurons to the physical cues generated by the geometry of the head and external ears. For sound sources located to one side of the midline, frequency-dependent interaural level differences (ILDs) may be generated by a combination of the spectral filtering effects produced by the external ears and the attenuation at the far ear due to the acoustic shadow cast by the head (Fig. 1A). In addition, the difference in path length from the sound source to each ear produces an interaural time difference (ITD) whose magnitude is determined by both the distance between the ears and the angle subtended by the source relative to the head (Fig. 1B).

Because of the tonotopic organization of the auditory system, these binaural comparisons mostly take place within frequency-specific channels. For simple periodic sounds, such as pure tones, the temporal fine structure is represented by the phase-locked discharges of auditory neurons at relatively low frequencies only (e.g., Sumner and Palmer, 2012). Moreover, interaural phase differences become spatially ambiguous as the sound frequency is increased (Mills, 1972, Blauert, 1997). Conversely, the ILDs generated by the shadowing effect of the head are the dominant localization cue when the wavelength of the sound is less than the distance between the two ears and therefore for adult humans at frequencies above ∼1700 Hz (Fig. 1A). This provides the basis for the duplex theory of sound localization (Strutt, 1907), whereby ITDs and ILDs are utilized for localizing low-frequency and high-frequency sounds, respectively.

**Fig. 2:**
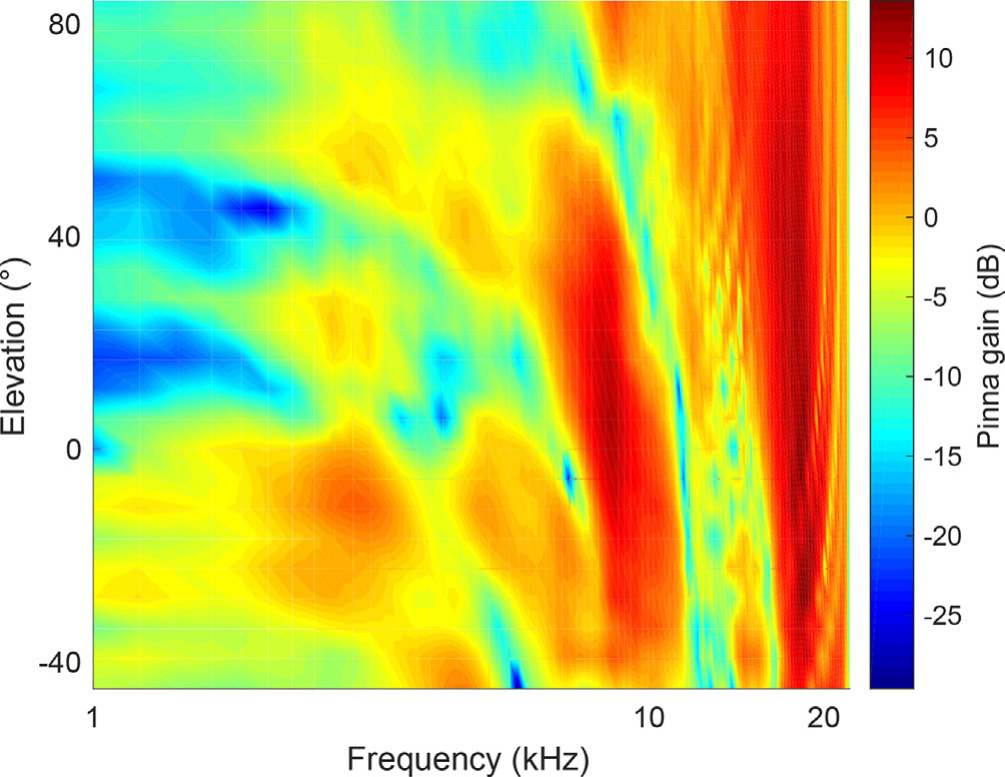
Monaural spectral cues to sound source elevation. Pinna gain for the right ear is shown as a function of sound frequency and elevation for sounds presented at 0° azimuth. Data are derived from head-related transfer function measurements published in the CIPIC database by Algazi et al. (2001). (Copyright (c) 2001 The Regents of the University of California. All Rights Reserved).

Because of their unusual ear asymmetry, barn owls are able to use the two binaural cues for localizing sounds at any angle relative to the head, relying on ILDs in the vertical plane while ITDs provide the principal basis for localization in the horizontal plane (Knudsen and Konishi, 1979). In most other species, however, the skull is symmetrical, with the values of both binaural cues varying predominantly in the horizontal plane. Vertical localization relies instead on spectral localization cues (Fig. 2), i.e., frequency-dependent changes in the level of the sound as the location of the source is varied (Blauert, 1997, Carlile et al., 2005). Spectral cues are also important in the horizontal plane as they provide the basis for determining whether sound sources are located in front or behind the listener, and therefore for resolving the cones of confusion that are inherent in the way binaural cues vary with spatial location (Blauert, 1997, Schnupp et al., 2011). Although these cues otherwise appear to contribute little to localization in the horizontal plane, which instead relies principally on ITDs and ILDs (Macpherson and Middlebrooks, 2002), we shall show in the following that the relative weighting of the cues used to localize sound sources in azimuth can change with experience, particularly following hearing loss in one ear.

In addition to providing a basis for localizing sounds, the ability to extract interaural information facilitates target detection in noisy environments (Darwin, 2006, Eramudugolla et al., 2008, Maddox and Shinn-Cunningham, 2012), a phenomenon known as spatial release from masking. This refers to the change in speech reception (or target detection) thresholds in the presence of interfering sounds when the target and masking sounds are spatially separated. Spatial release from masking is one process that can support auditory stream segregation, including the capacity to perceive a particular speaker's voice in “cocktail-party” situations, where other, interfering, sounds are simultaneously present (Cherry, 1953, Middlebrooks, 2017). The improvement in speech intelligibility that results from the spatial separation of the sound sources varies in magnitude with the nature of the masker, with a greater benefit being obtained with informational masking than with energetic masking (Arbogast et al., 2002).

Although spatial release from masking can occur in the absence of subjective sound localization, two key processes that support this phenomenon, better-ear listening and binaural unmasking, are explicitly dependent upon the interaural disparities that arise when a sound source is located to one side of the head. Better-ear listening can improve the signal-to-noise-ratio (SNR) at one ear for a target sound if the masker is attenuated due to the shadowing effect of the head. In realistic speech-in-noise scenarios, such as when multiple spatially-separated maskers are present, the better ear may fluctuate over time and frequency. Consequently, better-ear effects are thought to result from the auditory system's ability to “glimpse” these short-term changes in SNR (e.g., Brungart and Iyer, 2012, Schoenmaker and van de Par, 2017). In contrast to better-ear effects, binaural unmasking involves a comparison of information at the two ears (Licklider, 1948, Durlach, 1963). Detection thresholds for bilaterally presented pure tones in noise can be up to 15 dB lower when the phase of either signal is fully inverted in one ear, a measure known as the binaural masking level difference. Similar manipulations are known to improve the intelligibility of masked speech (measured using the binaural intelligibility level difference; Levitt and Rabiner, 1967).

Much less attention has been given to the role of spectral cues in spatial release from masking. Because the spectral filtering provided by the head, and particularly the external ears, is direction dependent, this will contribute to the better-ear effect at high sound frequencies. Indeed, there is some evidence that speech intelligibility in the presence of spatially-separated masking noise improves if natural spectral cues are available than when they are not (Rychtáriková et al., 2011). Nevertheless, both sound localization and spatial release from masking depend on binaural processing and will therefore be impaired, particularly if the target sound is located on that side, if hearing is lost in one ear.

## 3 Prevalence of unilateral hearing loss

Estimates of the prevalence of unilateral hearing loss, in which the impairment is restricted to one ear, vary with numerous factors, including the age of the subjects and, of course, the type and extent of the hearing loss (e.g. Bess et al., 1998, Lee et al., 2011, Berninger and Westling, 2011). For example, minimal sensorineural hearing loss in one ear (15–40 dB HL) has been reported in 3% of sampled school-age children (Bess et al., 1998). In terms of the potential impact on spatial hearing, it is just as important to consider asymmetric hearing loss, where both ears might be affected but to differing degrees. This is particularly the case in young children, where the changing incidence of either unilateral or bilateral otitis media with effusion with age is likely to provide highly variable experience of spatial cues for the majority of individuals (Hogan et al., 1997, Whitton and Polley, 2011). Furthermore, treatments for hearing loss may actually exacerbate asymmetric hearing, such as when individuals with severe to profound bilateral deafness receive a cochlear implant in one ear only or sequentially in the two ears. This may also be the case if there is a delay in providing a device to the affected ear when hearing loss is unilateral.

## 4 Effects of unilateral hearing loss on the developing auditory system

Experimental studies of the effects of unilateral hearing loss on spatial hearing have focussed primarily on the consequences of conductive loss (reviewed in Keating and King, 2013). It is important to note that sensorineural hearing loss can produce spectrotemporal processing deficits that would be expected to affect neural sensitivity to spatial localization cues (Moore, 1996, Felix and Portfors, 2007, Trujillo and Razak, 2013). Nevertheless, inducing a conductive hearing loss in one ear has the great advantage from an experimental perspective that it is, in principle, fully reversible. For example, monaural occlusion can be used in both humans and animals to produce a temporary imbalance in input between the two ears. From a clinical standpoint, understanding how the brain responds to conductive hearing loss can provide insight into the consequences of otitis media with effusion and other disorders that affect sound transmission through the external or middle ear.

A number of studies in animals have examined the effects of unilateral hearing loss during development on the morphology (Coleman and O'Connor, 1979, Webster and Webster, 1979, Moore et al., 1989), connectivity (Moore et al., 1989) and response properties (Clopton and Silverman, 1977, Silverman and Clopton, 1977, Moore and Irvine, 1981, Brugge et al., 1985, Popescu and Polley, 2010, Polley et al., 2013, Keating et al., 2013, Keating et al., 2015) of neurons at different levels of the auditory system. The results of many (though not all) of these studies are consistent with unilateral hearing loss causing a weakening of the representation of the deprived ear and a strengthening of the representation of the intact ear. Similarly, chronic stimulation of one ear via a cochlear implant during early life has been shown to result in a pronounced reorganization of cortical responses in humans (Gordon et al., 2013) and cats (Kral et al., 2013) in favor of the stimulated ear.

**Fig. 3:**
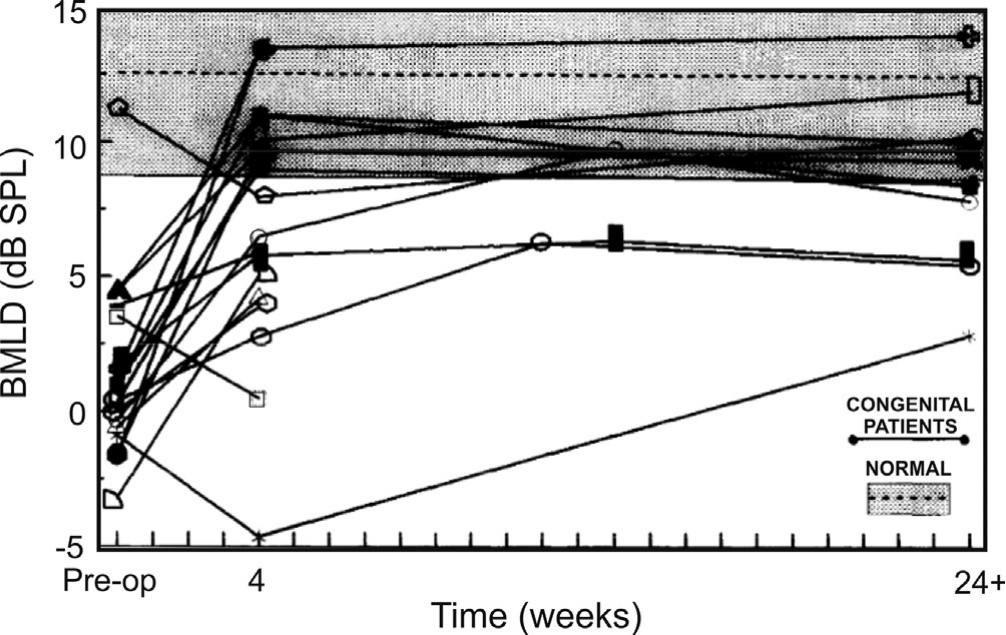
Binaural masking level difference (BMLD) in 19 patients before and after surgery to correct congenital unilateral hearing loss resulting from an abnormal external and/or middle ear on one side. The BMLD (N_0_S_0_ minus N_0_S_π_) is the difference in detection threshold of a tone presented either in phase or with the phase reversed between the ears in the presence of broadband noise, which was always presented in phase at the two ears. Some subjects had post-operative MLDs in the normal range, whereas others showed a persistent deficit in binaural processing. Modified with permission from Wilmington et al. (1994).

In terms of the consequences of unilateral or asymmetric hearing loss on spatial hearing, it is important to ask what effect this shift in aural preference has on neural sensitivity to binaural cues. Popescu and Polley (2010) addressed this by rearing rats with one ear canal ligated, which was reversed prior to carrying out electrophysiological recordings, and observed impaired binaural integration, with greater reorganization in the primary auditory cortex (A1) than in the inferior colliculus (IC). Furthermore, they found that this plasticity is more pronounced in infancy than in older animals. Other electrophysiological studies have also reported that abnormal binaural processing is present after correction of the unilateral hearing loss (Clopton and Silverman, 1977, Silverman and Clopton, 1977, Brugge et al., 1985) or following stimulation via bilateral cochlear implants (Tillein et al., 2016). The physiological changes induced by unilateral or asymmetric stimulation can be interpreted in terms of competitive interactions taking place in the developing brain between each ear. From a clinical perspective, they likely underpin the condition of amblyaudia or “lazy ear”, the persistent deficit in binaural processing experienced by people with a developmental history of asymmetric hearing loss (Snik et al., 1994, Kaplan et al., 2016). The consequences have been found to include impairments in sound localization and binaural unmasking, which can outlast restoration of function to the previously deprived ear (Clements and Kelly, 1978, Beggs and Foreman, 1980, Pillsbury et al., 1991, Wilmington et al., 1994, Moore et al., 1999, Gray et al., 2009) (Fig. 3).

Although these findings are indicative of maladaptive plasticity in binaural processing following unilateral hearing loss, other changes can take place that help to compensate for the impaired spatial hearing that would otherwise be observed. As previously mentioned, monaural spectral cues normally appear to contribute little to lateral location judgments (Macpherson and Middlebrooks, 2002). However, several studies have reported that some human listeners with single-sided deafness or severe-to-profound hearing loss in one ear can localize broadband or high-pass noise stimuli accurately in the horizontal plane (Newton, 1983, Slattery and Middlebrooks, 1994, Van Wanrooij and Van Opstal, 2004, Rothpletz et al., 2012, Agterberg et al., 2014, Firszt et al., 2017).

For example, Slattery and Middlebrooks (1994) compared the localization ability of monaural subjects who had congenital deafness in one ear with that of normal-hearing controls wearing a monaural earplug to simulate asymmetric hearing loss. Monaural occlusion in the controls severely disrupted sound localization in the horizontal plane, with the subjects displaying a large lateral response bias towards the open ear, and also affected vertical localization, particularly on the side of the plugged ear. In contrast, although two of the monaural patients tested gave similar results to the controls, the other three showed little or no lateral response bias and localized sounds on their deaf and hearing sides equally well. Slattery and Middlebrooks (1994) proposed that these listeners had learned to use the spectral cues of their intact ear to judge the lateral angle of a sound source, but also noted that the head-shadow effect may have influenced their performance.

Subsequent work in monaural listeners has confirmed this (Van Wanrooij and Van Opstal, 2004, Agterberg et al., 2014). Thus, relative to binaural controls, the horizontal localization judgments of monaural humans are much more affected by stimulus level, suggestive of a dependence on the attenuating effects of the head. Furthermore, in some cases, performance was found to be impaired by degrading spectral cues either by filling the concha of the intact ear with wax or by using low-frequency sounds where those cues provide little directional information. Monaural subjects appear to be quite variable, however, in their capacity to use spectral cues to localize in azimuth (Van Wanrooij and Van Opstal, 2004, Agterberg et al., 2014).

**Fig. 4:**
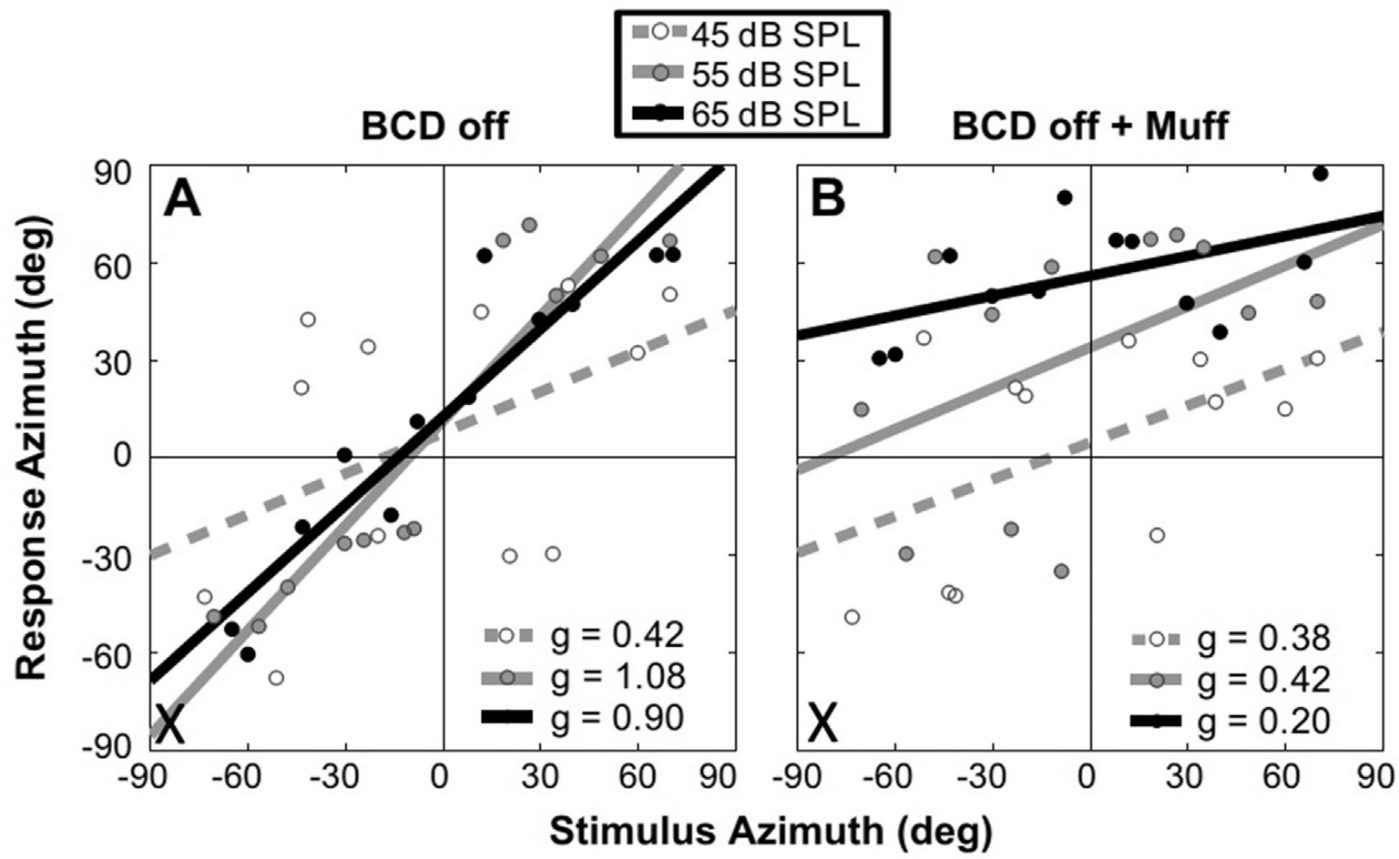
Unaided sound-localization responses for one subject with a unilateral conductive hearing loss in the left ear. The stimulus was broadband noise (0.5–20 kHz). These data were obtained without using the subject's bone-anchored device (BCD off) (A), and in the BCD off condition with an additional muff over the impaired ear to further alter binaural cues (B). The gains of responses (obtained from the slopes of the regression lines fitted to the data) to stimuli with levels of 55 dB SPL (solid gray regression lines) and 65 dB SPL (solid black regression lines) decreased significantly when the impaired ear was covered with the muff, indicating that the subjects were relying on binaural cues for localization, whereas this was not the case at the lower level of 45 dB SPL (gray dashed regression lines), which was unlikely to be audible at the deprived ear. g = response gain. Reproduced with permission from Agterberg et al. (2012).

Apart from individuals with total deafness in one ear, an important consideration is whether plasticity in the processing of spectral cues can enhance the localization accuracy of subjects with partial hearing loss and who may therefore have access to binaural cues that provide conflicting spatial information. Human listeners with a normal history of binaural hearing during childhood who then experience impaired hearing in one ear, due either to acquired conductive hearing loss (Agterberg et al., 2012) or the presence of an earplug (Van Wanrooij and Van Opstal, 2007), can use spectral cues to localize low-level broadband sounds that are insufficiently loud to reach the affected ear. However, based on the degradation in performance observed at higher sound levels when the input to the impaired ear is further reduced by covering it with a muff, Agterberg et al. (2012) concluded that listeners with acquired unilateral conductive hearing loss are also able to use their abnormal binaural cues to localize sounds in azimuth (Fig. 4).

The capacity of the developing auditory system to compensate for asymmetric hearing loss by becoming more dependent on the spectral cues generated by the intact ear has been demonstrated most clearly by rearing ferrets with one ear occluded with earplugs that attenuated acoustical inputs by 15–45 dB in a frequency-dependent fashion and delayed them by ∼110 μs (Keating et al., 2013, Keating et al., 2016). The use of an animal model affords more control over the age of onset and duration of the hearing loss than is possible in studies in people. In these experiments, monaural occlusion began at around 4 weeks of age, corresponding to the onset of auditory function in this altricial species, and continued for several months until the animals were fully grown. During this time, brief intermittent periods of normal hearing were provided by removing the earplug, in order to more closely mimic the fluctuating periods of hearing loss associated with otitis media with effusion.

**Fig. 5:**
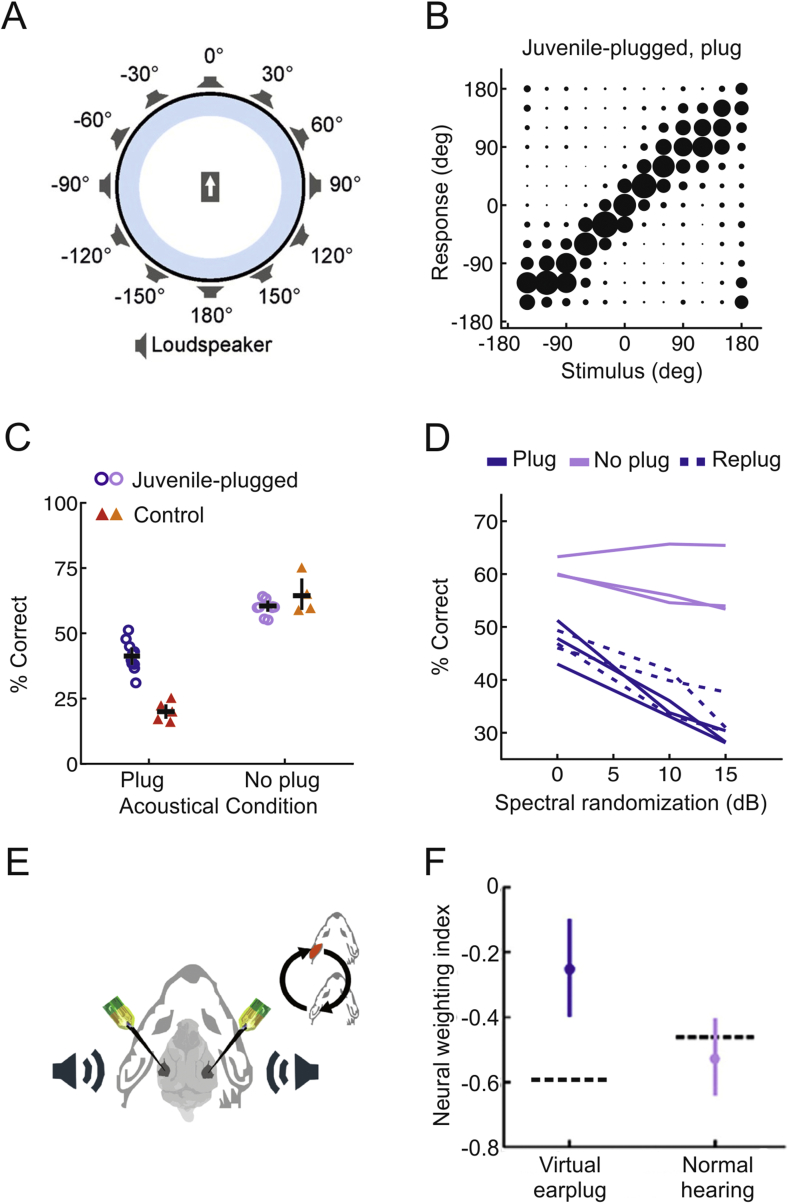
Adaptation to asymmetric hearing loss during infancy can be achieved by reweighting auditory spatial cues. (A) Schematic of setup used for measuring sound localization in the horizontal plane by adult ferrets. Twelve loudspeakers were located at 30° intervals around the perimeter of the apparatus. 0° is straight ahead, with negative numbers indicating locations to the animal's left. A trial was initiated by the animal licking a spout at the center of the chamber. This triggered the presentation of a burst of broadband noise with a flat spectrum from one of the loudspeakers; the animal received a water reward for making a correct approach-to-target response. (B) Average joint distributions of stimulus and response location for ferrets raised wearing an earplug in the left ear (interspersed with brief periods of normal hearing); the size of the circles represents the proportion of trials for each stimulus-response combination. These data were obtained with the earplug in place; note the similarity in the accuracy of the localization responses on the plugged and non-plugged sides. (C) Percentage correct scores for control and juvenile-plugged groups, with individual animals denoted by symbols. Horizontal lines indicate mean values, with error bars showing bootstrapped 95% confidence intervals. Acutely plugging one ear (‘Plug’) in the normally-raised control ferrets caused a substantial drop in localization accuracy. Significantly higher scores were achieved by the juvenile-plugged ferrets, and these animals localized just as accurately as the control group when the earplug was removed (‘No plug’). (D) Effect of disrupting spectral cues by increasing the degree of spectral randomization in the stimuli on localization accuracy by juvenile-plugged animals with and without an earplug in place. (E) Recordings were made bilaterally in the primary auditory cortex (A1) of these animals. (F) Neurons in juvenile-plugged animals were more sensitive to the monaural spatial cues provided to the intact ear and less sensitive to the other available cues; this is indicated by the higher weighting index (mean ± 95% confidence intervals) in juvenile-plugged animals than in the control group (whose mean values are indicated by the horizontal dashed lines). Increased weighting of spectral cues in juvenile-plugged animals was observed only when a virtual earplug was introduced to the previously occluded ear during the recordings. Adapted with permission from Keating et al. (2013).

The animals were trained to perform a free-field sound localization task in which noise bursts were presented from one of 12 loudspeakers positioned at 30° intervals around the perimeter of the testing chamber (Fig. 5A). The performance of the animals was assessed as the duration, level and spectral composition of the stimulus were varied, by measuring both the accuracy and latency of the initial head orienting response made following sound presentation and the loudspeaker/reward spout subsequently approached. As expected, acute monaural occlusion in normally-reared control animals resulted in an immediate decline in localization accuracy (Fig. 5C). However, ferrets raised with an earplug placed in one ear and tested with that ear still occluded were able to localize broadband sounds reasonably well at all locations tested, indicating that the developing auditory system had adapted to a substantial degree to the asymmetric hearing loss (King et al., 2000, Keating et al., 2013, Keating et al., 2015) (Fig. 5B and C).

Following removal of the earplug, these animals were able to localize sounds as accurately as the controls (Fig. 5C). This lack of an after-effect argues against the basis for adaptation being a systematic remapping of sensitivity to the altered binaural cues. To examine the role of pinna cues at the non-occluded ear, Keating et al. (2013) randomized the spectrum of the broadband noise bursts across trials so that it was not possible to determine whether spectral features were due to the filtering effects of the head and ears or were instead properties of the stimulus itself. When tested with the ear plugged, sound localization performance in ferrets raised with one ear occluded declined as the amount of spectral randomization was increased, but this effect largely disappeared once the earplug was removed (Fig. 5D). In other words, the animals' horizontal localization behavior was guided by spectral cues in the asymmetric hearing loss condition, but not when normal binaural inputs were available. This was confirmed by calculating the mean stimulus spectrum preceding responses to each of the 12 loudspeaker locations, which revealed high-frequency spectral features that matched the directional transfer function of the intact ear. Electrophysiological recordings from these animals showed that A1 neurons carried more information about the spectral cues available at the non-occluded ear, but again only when a conductive hearing loss was applied to the previously occluded ear (Keating et al., 2013) (Fig. 5E and F).

**Fig. 6:**
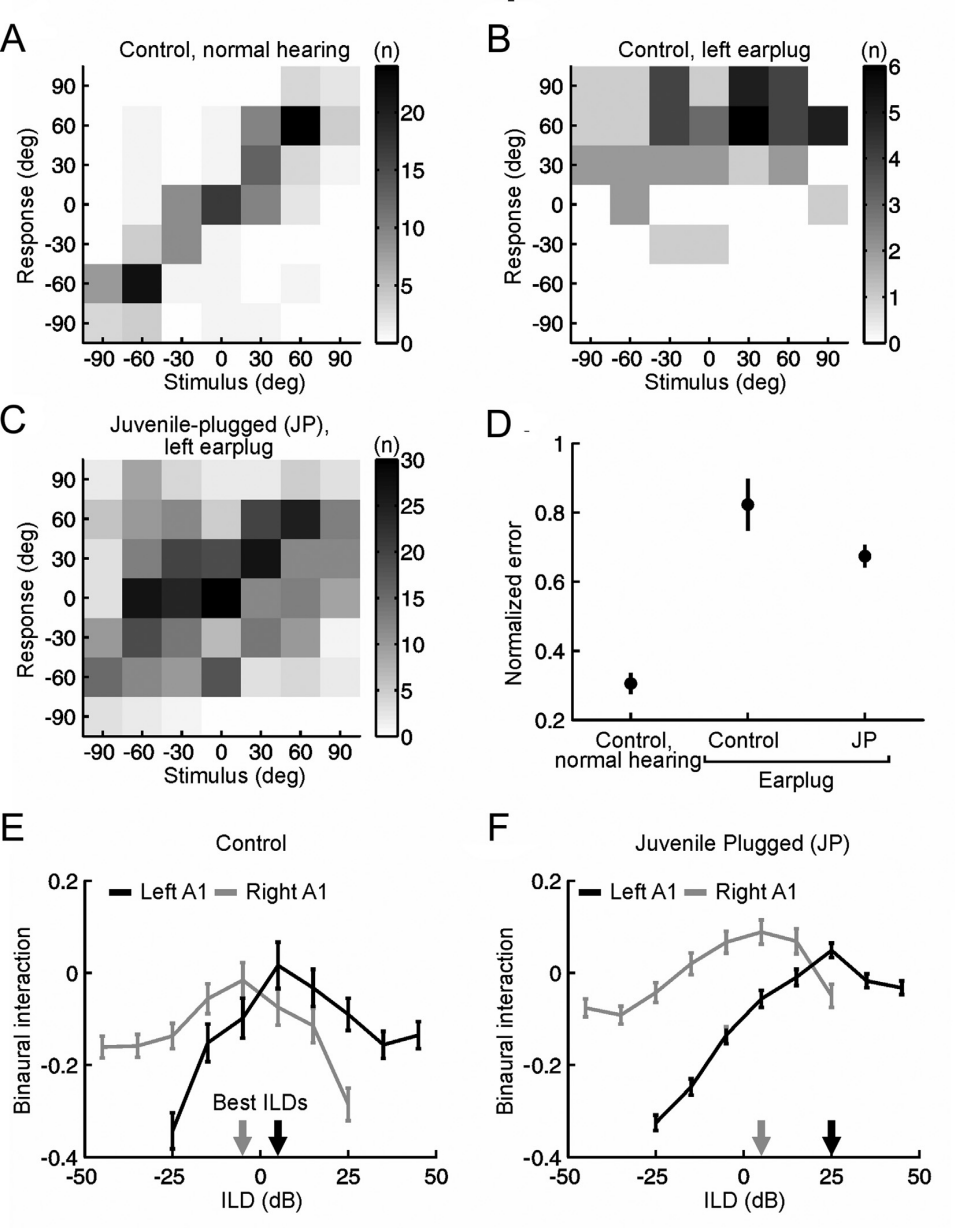
Adaptation to asymmetric hearing loss during infancy by remapping the altered binaural cues onto new locations in space. (A–C) Joint distributions of stimulus and response, expressed as degrees (deg) azimuth, for a control ferret with normal hearing (A) and a control (B) and juvenile-plugged (JP) ferret (C) wearing an earplug in the left ear. Grayscale represents the number of trials (n) corresponding to each stimulus-response combination. (D) Mean unsigned error for control and earplugged ferrets, normalized so that 0 and 1 correspond to perfect and chance performance, respectively. Error bars show bootstrapped 95% confidence intervals. Controls wearing an earplug (n = 6 ferrets) made larger errors than normal hearing controls (n = 4; P < 0.001, bootstrap test). While wearing an earplug, juvenile-plugged ferrets (n = 2) made smaller errors than acutely plugged controls (P < 0.001, bootstrap test). (E) Mean binaural interaction (±s. e.m.) as a function of ILD across neurons recorded in A1 of control ferrets under normal hearing conditions. Data are plotted separately for left (n = 142 units, black) and right (n = 177 units, gray) A1. Best ILDs for each hemisphere are indicated by arrows. (F) Binaural interaction functions (mean ± s. e.m.) in juvenile-plugged ferrets under normal hearing conditions, which are shifted, relative to controls, in the appropriate direction to compensate for the hearing loss experienced during development. Adapted with permission from Keating et al. (2015).

This strategy of up-weighting spectral cues in a context-dependent fashion following a history of asymmetric hearing loss enables accurate sound localization to be maintained irrespective of whether the hearing loss is present or not in the other ear. In fact, the auditory system possesses an even greater capacity for accommodating abnormal spatial cues. If access to spectral localization cues is minimized by using narrowband noise bursts as stimuli, ferrets raised with one ear occluded still exhibit adaptive plasticity in both their behavioral and cortical responses, but this is now achieved via a partial compensatory adjustment in ILD sensitivity (Keating et al., 2015) (Fig. 6). Both forms of adaptation can be observed in the same animals, with largely separate populations of A1 neurons showing adaptive plasticity in the processing of monaural spectral cues and binaural cues (Keating et al., 2016).

It is unclear whether providing these animals with intermittent episodes of normal hearing while they were being raised with one ear occluded is required for the observed adaptation in their spatial hearing abilities. However, it has been shown that providing cats with brief periods of binocular vision during development can reduce the amblyopia, or loss of visual acuity, that would otherwise result from monocular deprivation (Mitchell et al., 2003, Mitchell et al., 2011). It is therefore possible that some experience of normal hearing during development may be necessary if spatial hearing abilities are to be preserved following asymmetric hearing loss, which has implications for the timing of treatment in children with hearing disorders (Gordon et al., 2013, Keating and King, 2013).

## 5 Adaptation to unilateral hearing loss in the mature auditory system

Reversible manipulation of acoustic localization cues has been widely used to probe the adaptive capabilities of the auditory system in adulthood. Plasticity during development is clearly important for calibrating neural circuits during the period when these cues are naturally changing in value as the head and ears grow (Schnupp et al., 2003). It also provides a potential means of adjusting to recurring periods of hearing loss that may be experienced during infancy (Hogan et al., 1997, Whitton and Polley, 2011). Although studies in barn owls (Knudsen et al., 1984, Knudsen, 1985) and rodents (Popescu and Polley, 2010, Polley et al., 2013) have shown that changes in binaural processing in response to asymmetric hearing loss are restricted to, or at least most pronounced during, a sensitive period of development, there is now overwhelming evidence in mammals that the adult brain can also learn to utilize abnormal spatial cues (reviewed by Mendonça, 2014).

Several studies have shown that horizontal localization by adult humans can adapt to varying degrees to asymmetric hearing loss induced by occluding one ear, resulting in a partial recovery in their ability to localize sound (Bauer et al., 1966, Butler, 1987, Kumpik et al., 2010, Irving and Moore, 2011, Keating et al., 2016). An important question is whether this plasticity is driven solely by training on the localization task or whether other factors contribute. Although listeners with normal hearing can learn within a few hours to reinterpret the relationship between auditory localization cues and directions in space (e.g., Mendonça et al., 2013, Shinn-Cunningham et al., 1998, Zahorik et al., 2006), the spacing of the trials seems to be important for adaptation to hearing loss in one ear (Musicant and Butler, 1980, Kumpik et al., 2010). For example, Kumpik et al. (2010) observed steady improvements in performance in subjects who wore an earplug all day (except during showering or sleep) if the sound localization training was distributed across several days, but not in a second group who completed a similar number of trials compressed into one day. This implies that a period of memory consolidation may be required for adaptation to asymmetric hearing loss.

Experiments in monaurally-plugged adult ferrets have shown that the extent of the recovery in localization accuracy is determined by the frequency of training (Kacelnik et al., 2006). These animals adapted more quickly and more extensively when provided with daily training than when the training sessions were more spread out, even though the same overall number of trials were included. The ferret experiments also demonstrated that the improvements in localization accuracy were specific to auditory training, and that neither vision nor feedback about the accuracy of the response were required for some adaptation to take place (Kacelnik et al., 2006). It is likely, however, that other sensory, motor and cognitive factors may promote learning (Strelnikov et al., 2011, Carlile et al., 2014) when abnormal auditory cues are experienced. For example, a greater improvement in auditory localization accuracy has been observed in human listeners wearing an earplug if performance feedback is provided, and especially if the auditory stimuli are accompanied by spatially-congruent visual cues (Strelnikov et al., 2011).

**Fig. 7:**
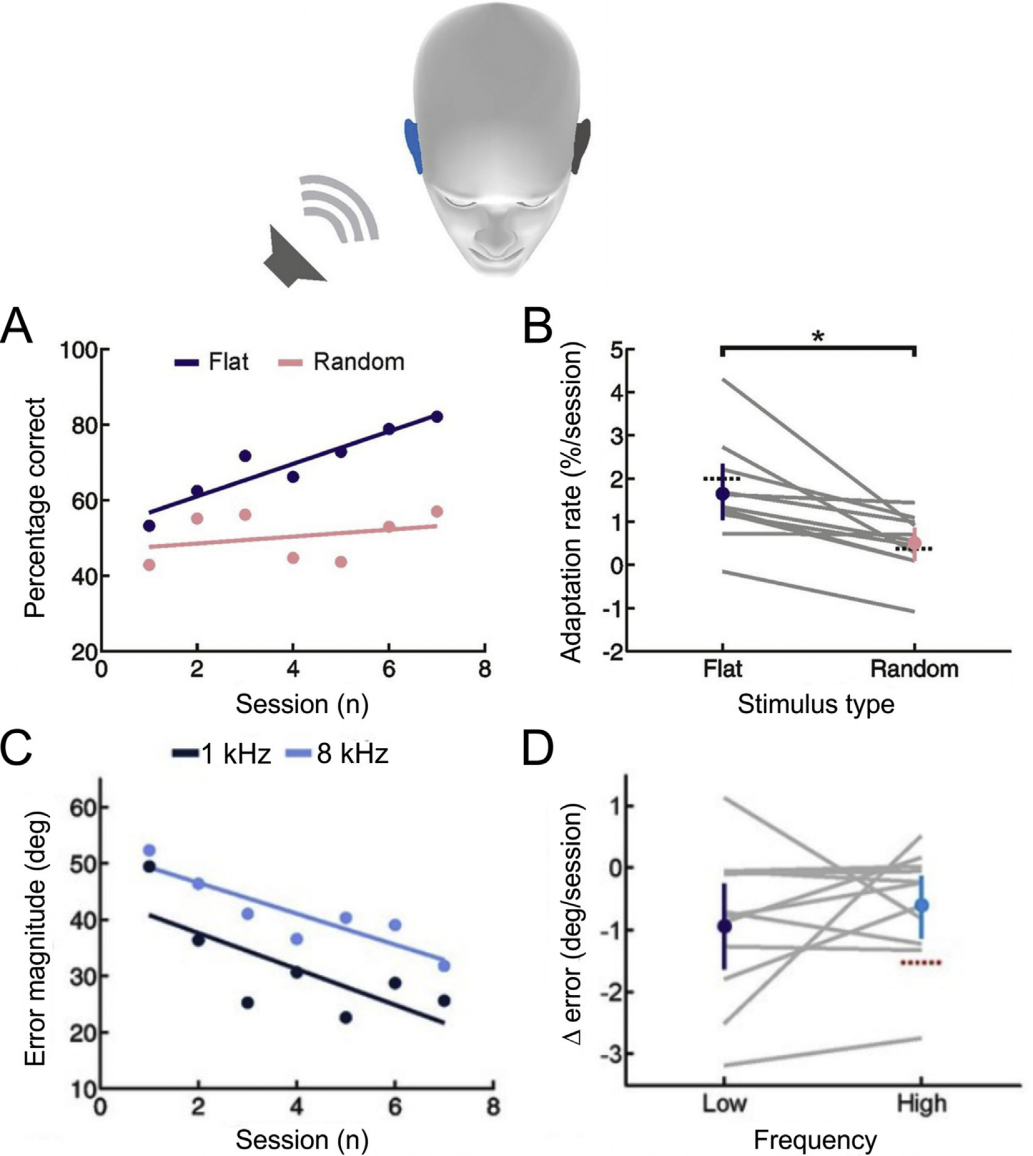
Adult human listeners can relearn to localize sound after introducing an asymmetric hearing loss by occluding one ear. (A) Sound localization performance (% correct) as a function of training session for one subject who wore an earplug in the right ear during the localization tests. Scores for each session (dots) were fitted using linear regression (lines) to calculate slope values, which quantified the rate of adaptation. Relative to flat-spectrum noise (blue), much less adaptation occurred with random-spectrum noise (pink), which limits the usefulness of spectral cues to sound location. (B) Adaptation rate is shown for flat- and random-spectrum stimuli for different subjects (gray lines; n = 11). Positive values indicate improvements in localization performance with training. Mean adaptation rates across subjects (±bootstrapped 95% confidence intervals) are shown in blue and pink for the two stimulus types. Dotted black lines indicate adaptation rates observed in a previous study (Kumpik et al., 2010). (C) Mean error magnitude plotted as a function of training session for one subject when pure tones were used as the stimuli. Data are plotted separately for low- (1 kHz, dark blue) and high-frequency (8 kHz, light blue) tones. Improved performance was associated with a reduction in error magnitude, producing negative values for the change (Δ) in error magnitude. (D) Δ error for low- and high-frequency tones plotted for each subject (gray lines; n = 11). Mean values for Δ error across subjects (±bootstrapped 95% confidence intervals) are shown in blue. Although there are pronounced individual differences for the adaptation observed at the two tone frequencies, almost all values are <0, indicating that error magnitude declined over the training sessions. Dotted red line shows Δ error values that would have been observed if human listeners had adapted as well as ferrets reared with a unilateral earplug (Keating et al., 2015). Adapted from Keating et al. (2016).

As with monaural deprivation during development, when broadband sounds are used as stimuli, adaptation of auditory localization behavior to asymmetric hearing loss in adulthood is based on subjects learning to rely more than before on the unchanged spectral localization cues provided by the normal ear (Kacelnik et al., 2006, Kumpik et al., 2010, Keating et al., 2016) (Fig. 7A and B). These findings therefore support the growing body of evidence from studies in which spectral localization cues are altered by mechanically reshaping the external ear (Hofman et al., 1998, Van Wanrooij and Van Opstal, 2007, Carlile et al., 2014, Trapeau et al., 2016, Watson et al., 2017) or by presenting virtual acoustic space stimuli using non-individualized head-related transfer functions (Zahorik et al., 2006, Parseihian and Katz, 2012) for considerable plasticity in the way these cues are processed in the brain.

Nevertheless, reweighting of different spatial cues is not the only means of learning to localize sounds accurately when one ear is occluded. Perceptual learning studies carried out in listeners with normal hearing have shown that sensitivity to binaural spatial cues can improve with training (Wright and Fitzgerald, 2001, Kumpik et al., 2009, Sand and Nilsson, 2014) and, as mentioned in the previous section, binaural plasticity represents part of the basis for adaptation to asymmetric hearing loss during infancy. Although Kumpik et al. (2010) found no changes in ITD or ILD sensitivity over a week long period of monaural occlusion in adult humans, during which performance on a free-field localization task gradually improved, exposing subjects to altered cues only during training sessions did result in remapping of both binaural cues onto appropriate locations (Keating et al., 2016) (Fig. 7C and D). Thus, different strategies for recovering sound localization accuracy in the presence of asymmetric hearing loss are also present in adulthood, with individual subjects varying in the extent to which they adapted by cue reweighting or cue remapping (Keating et al., 2016).

The behavioral plasticity observed in ferrets raised with a unilateral conductive hearing loss is mirrored by changes in the processing of sound localization cues in A1 (Keating et al., 2013, Keating et al., 2015). However, adaptive changes in the auditory spatial tuning of neurons in the superior colliculus have also been described in monaurally-deprived animals (King et al., 1988, King et al., 2000), so the site of plasticity remains unclear. The ability of adult ferrets to compensate with training to temporary loss of hearing in one ear requires a functioning auditory cortex (Nodal et al., 2012), but is also impaired if the layer V neurons in A1 that project to the IC are selectively eliminated (Bajo et al., 2010). Thus, although the auditory cortex plays a critical role in spatial hearing and in the experience-dependent plasticity that allows the brain to compensate for asymmetric reversible hearing loss, its descending projections appear to play a specific role in retraining the auditory system.

## 6 Perceptual training for hearing-impaired listeners

Compared with the large body of work that has examined the effects of sound localization training in normal-hearing listeners with unperturbed or perturbed hearing, attempts to translate such training to clinical populations have only recently gathered pace. This is perhaps because of a prior emphasis on providing speech recognition training (Henshaw and Ferguson, 2013, Fu et al., 2015), and also possibly because the potential benefits of functional plasticity in bilateral and bimodal artificial or amplified hearing for spatial masking release and sound localization have only recently started to be recognized. Recently, Firszt et al. (2015) provided a rich free-field sound localization training regime with visuospatial feedback and showed that adults with severe to profound unilateral hearing loss can be trained to more accurately localize broadband sounds with complex spectral and temporal structure in the horizontal plane, and that this improvement generalized to the localization of monosyllabic words. This finding is somewhat analogous to those of the monaural ear-plugging studies described above with normal-hearing listeners, although the relative contribution of changes in the weights given to head shadow versus spectral cues was not quantified in that study, and so the extent to which the improvements might generalize to real-world spatial scenes is unclear.

Given the considerable evidence from earplugging studies that a key step in compensating for an imbalance in inputs between the two ears is to change the weighting of different spatial cues, with the auditory brain becoming more dependent on the unchanged spectral cues available at the non-affected ear, it is important to ask how clinically relevant this might be. It is clear that at least some people who are deaf in one ear do indeed utilize monaural spectral cues for localization in the horizontal plane (e.g. Newton, 1983, Slattery and Middlebrooks, 1994, Van Wanrooij and Van Opstal, 2004). Furthermore, the improvement in sound localization sometimes reported in blind individuals has been attributed to their greater sensitivity to spectral cues corresponding to lateral sound locations (Doucet et al., 2005, Voss et al., 2011), providing further evidence for compensatory plasticity in the use of spectral localization cues.

However, whether listeners provided with hearing devices can benefit in the affected ear in a similar fashion is more questionable. For one thing, the progressive loss of high-frequency hearing in age-related sensorineural hearing loss will restrict the availability of spectral cues. Although modern hearing aids can have bandwidths of up to 10 kHz or more (Kuk and Baekgaard, 2009), which can help hearing-impaired subjects to understand target speech in the presence of spatially-separated masking speech (Carr Levy et al., 2015), spectral cues are seriously distorted by the use of microphones that do not sit inside the auditory canal, such as in behind-the-ear hearing aids (Moore and Popelka, 2016). There is some indication that inclusion of algorithms that preserve pinna cues can improve horizontal localization and speech perception in noise in hearing aid users (Kuk et al., 2013, Xu and Han, 2014, Korhonen et al., 2015, Gomez and Seeber, 2017). However, this will depend on whether the hearing aids provide sufficient amplification for individual listeners at the high frequencies where most of the directional information is available in these cues.

The finding that listeners with one ear plugged can be trained to partially recover their sound localization accuracy by learning to remap the distorted ILDs and ITDs onto appropriate spatial locations (Keating et al., 2016) potentially offers much greater scope for utilizing adaptive plasticity to promote improvements in spatial hearing in the hearing impaired. Indeed, the results of this study raise the possibility of adopting targeted training strategies based on the residual hearing abilities of individual patients and therefore the localization cues they have available. This is also relevant to patients with cochlear implants whose limited spatial hearing abilities can be improved if they adapt their ILD sensitivity to the range of values provided by the output of the implants (Dorman et al., 2014; Dorman et al., 2015) and by enhancing the availability of ILDs at low frequencies (Brown, 2014). Recent studies have started to examine the effects of training on cochlear implant users who have either been implanted bilaterally or retain access to binaural information as a result of having one good ear. There is some indication that sound localization training can promote binaural hearing, both with unilateral implantation when the other ear is preserved (Nawaz et al., 2014) and with bilateral cochlear implants (Tyler et al., 2010). Furthermore, a training paradigm in which auditory and visual stimuli were randomly interleaved has been shown to improve the auditory localization accuracy and cortical coding of ILDs in adult ferrets fitted with bilateral cochlear implants following deafening in infancy (Isaiah et al., 2014).

## 7 Conclusions

The studies discussed in this review have demonstrated that the auditory system can adjust to changes in the available sound localization cues in ways that can help to preserve spatial hearing abilities. This can be achieved either by reweighting different cues, as demonstrated by the evidence for greater reliance on spectral cues when ITDs and ILDs are compromised by unilateral hearing loss, or by learning a new relationship between altered binaural cues and sound source location. Utilizing the remarkable plasticity of auditory localization mechanisms in the treatment of clinical populations will require the development of training protocols that are practical to use outside the laboratory and which confer maximum generalization to regions of space and stimulus types other than those used for training. This includes identifying the type of feedback most likely to promote learning, with recent studies suggesting that sensorimotor feedback can improve the rate and extent of adaptation to altered spatial cues (Carlile et al., 2014, Keating et al., 2016). Most importantly, if plasticity in the neural processing of auditory spatial cues is to have therapeutic value, it will be necessary to show that it extends to more realistic and challenging listening situations than those typically used in the laboratory, and that the benefits include not only a recovery in sound localization accuracy, but also improved speech-in-noise perception.
